# *TES* functions as a Mena-dependent tumor suppressor in gastric cancer carcinogenesis and metastasis

**DOI:** 10.1186/s40880-019-0347-y

**Published:** 2019-02-06

**Authors:** Dan-dan Wang, Yi-bing Chen, Jing-jing Zhao, Xiao-fei Zhang, Guang-chao Zhu, De-sheng Weng, Ke Pan, Lin Lv, Qiu-zhong Pan, Shan-shan Jiang, Lei-lei Wang, Jian-chuan Xia

**Affiliations:** 1 Sun Yat-sen University Cancer Center; State Key Laboratory of Oncology in South China, Collaborative Innovation Center for Cancer Medicine; Guangdong Key Laboratory of Nasopharyngeal Carcinoma Diagnosis and Therapy, Guangzhou, 510060 Guangdong P.R. China; 2 Department of Biotherapy, Sun Yat-sen University Cancer Center, 651 Dongfeng Road East, Guangzhou, 510060 Guangdong P.R. China; 3 Shandong Medicinal Biotechnology Centre, Back and Neck Pain Hospital, Shandong Academy of Medical Sciences, Jinan, 250062 Shandong P.R. China; 4 Genetic and Prenatal Diagnosis Center, Department of Gynecology and Obstetrics, First Affiliated Hospital of Zhengzhou University, Zhengzhou, 450052 Henan P.R. China; 5 Key Laboratory for Applied Microbiology of Shandong Province, Ecology Institute of Shandong Academy of Sciences, Jinan, 250014 Shandong P.R. China

**Keywords:** Gastric cancer, *TES*, Tumor suppressor, Mena, Lamellipodin

## Abstract

**Background:**

In our previous study, we identified a candidate tumor suppressor gene, testin LIM domain protein (*TES*), in primary gastric cancer (GC). TES contains three LIM domains, which are specific interacting regions for the cell adhesion and cytoskeleton regulatory proteins. Mena is a known cytoskeleton regulator that regulates the assembly of actin filaments and modulates cell adhesion and motility by interacting with Lamellipodin (Lpd). Therefore, we hypothesized that TES plays a role as tumor suppressor in GC through interacting with Mena. This study aimed to investigate the tumor suppressive functions of TES in GC.

**Methods:**

We explored the tumor suppressive effect of TES in GC by in vitro cell proliferation assay, colony formation assay, cell cycle analysis, Transwell assays, and in vivo tumorigenicity and metastasis assays. The interaction of TES and Mena was investigated through immunoprecipitation-based mass spectrometry. We also analyzed the expression of TES and Mena in 172 GC specimens using immunohistochemistry and investigated the clinicopathological and prognostic significance of TES and Mena in GC.

**Results:**

TES suppressed GC cell proliferation and colony formation, induced cell cycle arrest, and inhibited tumorigenicity in vitro. Additionally, it inhibited GC cell migration and invasion in vitro and suppressed metastasis in vivo. TES interacted with Mena, and inhibited the interaction of Mena with Lpd. Transwell assays suggested that TES suppressed migration and invasion of GC cells in a Mena-dependent fashion. In GC patients with high Mena expression, the expression of TES was associated with tumor infiltration (*P* = 0.005), lymph node metastasis (*P* = 0.003), TNM stage (*P* = 0.003), and prognosis (*P* = 0.010). However, no significant association was observed in GC patients with low Mena expression.

**Conclusions:**

We believe that TES functions as a Mena-dependent tumor suppressor. TES represents a valuable prognostic marker and potential target for GC treatment.

**Electronic supplementary material:**

The online version of this article (10.1186/s40880-019-0347-y) contains supplementary material, which is available to authorized users.

## Background

Gastric cancer (GC) is a common malignancy worldwide, with about one million new cases each year [1]. Despite observable advances in surgical techniques, chemotherapy, and radiotherapy, the prognosis of GC patients remains unsatisfactory [2]. Metastasis is one of the leading causes of death in GC patients [2]. Carcinogenesis and metastasis of GC are multistep processes, involving activation of oncogenes and inhibition of tumor suppressor genes (TSGs). Therefore, understanding the molecular mechanisms underlying carcinogenesis and metastasis of GC is vital.

In our previous study, we identified a candidate TSG, testin LIM domain protein (*TES*), at D7S486 on 7q31.1/2 in primary GC tissues due to its high frequency of loss of heterozygosity [3]. Furthermore, we found that the *TES* promoter was frequently hypermethylated in primary GC tissues and GC cell lines, and the protein expression of TES was significantly decreased in 72% GC tissues as compared with matched non-tumor tissues [3]. TES is predicted to encode a highly conserved protein of 421 amino acids containing three C-terminal LIM domains [4]. LIM domains each consists of two zinc-finger motifs that mediate protein–protein interactions with transcription factors, cytoskeletal proteins, and signaling proteins [4–6]. TES has been identified as a putative TSG in many human cancers, such as breast and uterine cancers [7] and glioblastoma [8]. In these cancer types, the expression of TES was decreased or totally lost by promoter hypermethylation [7, 8]. Overexpression of TES significantly inhibited tumor cell growth in vitro and reduced the tumorigenic potential of certain tumor cell lines in vivo [7]. Moreover, *TES* knockout in mice resulted in increased susceptibility to carcinogen-induced GC [9]. However, the role of TES in GC has not been further investigated, and the molecular mechanism of TES underlying GC carcinogenesis and metastasis remains unknown.

Previous studies have shown that TES localized to focal adhesions and cell–cell or cell–substratum contact sites, suggesting a role in cell adherence, migration, and motility [4, 10, 11]. In addition, it is an interacting partner of the known cell adhesion and cytoskeleton regulatory proteins, such as Zyxin, Talin, and Mena [4, 5]. Mena, a member of the Ena/vasodilator-stimulated phosphoprotein (VASP) family, is involved in regulating the assembly of actin filaments and modulates cell adhesion and motility [5, 12–14]. Ena/VASP family proteins can recruit MRL proteins (consisting of Mig10, Rap1-interacting adapter molecule [RIAM], and Lamellipodin [Lpd]) to the leading edge of filopodia and lamellipodia to regulate cell lamellipodial spreading and motility [5, 15]. It has been reported that Mena is involved in cell migration and motility by its interaction with Lpd [15]. Therefore, we hypothesized that TES plays a role as tumor suppressor in GC through interacting with Mena.

In this study, we systematically explored the tumor suppressive functions of TES in GC both in vitro and in vivo and determined its interaction with Mena in GC.

## Materials and methods

### Cell lines

All cell lines were authenticated by short-tandem repeat analysis. The human embryonic kidney cell line HEK293A (obtained in November 2009, authenticated in June 2015) and GC cell lines MKN45, SGC7901, MGC803, AGS, and HGC27 (obtained in July 2011, authenticated in June 2015) were obtained from the Committee of Type Culture Collection of Chinese Academy of Sciences (Shanghai, China). All cells were cultured in RPMI-1640 medium supplemented with 10% fetal bovine serum (FBS) at 37 °C in a humidified chamber containing 5% CO_2_.

### Patients and tissue samples

The medical records of 172 GC patients treated at Sun Yat-sen University Cancer Center (Guangzhou, China) between January 2003 and December 2005 were reviewed. The patient selection criteria were as follows: (1) the patient was pathologically diagnosed with gastric adenocarcinoma; (2) the patient had received gastrectomy with limited or extended lymphadenectomy; (3) the patient did not receive any anticancer treatment before surgery; (4) the patient had complete clinical information, including follow-up data; (5) the patient had no other synchronous malignancies or familial malignancy; (6) the patient had no recurrent or remnant GC; and (7) the patient survived at least 3 months after surgery. Follow-up data were obtained through on-site interview, telephone calling or medical chart review. Overall survival (OS) was defined as the time from surgery to death from any cause or last follow-up. The study was approved by the Ethics Committee of Sun Yat-sen University Cancer Center (Guangzhou, China), and written informed consent was obtained from all participants.

### Recombinant adenoviral expression vector construction and transfection

The TES recombinant adenoviral expression vector (Ad-TES) and control vector (Ad-Control) were constructed using the Gateway cloning system (Invitrogen, Carlsbad, CA, USA), according to the manufacturer’s protocol. After linearization by PacI enzyme, Ad-TES and Ad-Control were transfected into HEK293A cells using Lipofectamine 2000 (Invitrogen). After 10–13 days, when an approximately 80% cytopathic effect was observed, cells and medium were collected. After lysing the cells by three freeze–thaw cycles, the adenoviral supernatant was harvested by centrifugation (1000×*g*) at 4 °C for 15 min, tittered using Adenovirus Titer Immunoassay Kit (Innogent, Shenzhen, Guangdong, China) and stored at − 80 °C. To increase the transfection efficiency, HEK293A cells were re-infected with the initial harvested viral supernatant. Three to 4 days later, the cell lysates were collected after three freeze–thaw cycles. MKN45 or SGC7901 cells were transfected with Ad-TES at a multiplicity of infection (MOI) of 200. The transfection efficiency was calculated by dividing the amount of cells presenting green fluorescence by the total number of attached cells in 10 fields randomly selected for each sample under a fluorescence microscope with 100× magnification.

### Extraction of total RNA and reverse transcription-polymerase chain reaction (RT-PCR)

Total RNA was extracted using TRIzol (Invitrogen) according to the manufacturer’s protocol. The concentration of total RNA was assessed by measuring absorbance at 260 nm using a NANO DROP spectrophotometer (ND-1000, Thermo Scientific, Waltham, MA, USA). Two mg of total RNA was reversely transcribed into cDNA using M-MLV reverse transcriptase (Promega, Madison, Wisconsin, USA) according to the manufacturer’s recommendation. The cDNA templates were amplified using the specific primer set for TES. The samples amplified with glyceraldehyde-3-phosphate dehydrogenase (GAPDH) primer set were used as an internal control. Primers used in this study were as follows: the forward primer 5′-CATGGACCTGGAAAACAAAGTG-3′ and the reverse primer 5′-CTAAGACATCCTCTTCTTACATTCCAC-3′ for TES; the forward primer 5′-CGGGAAGCTTGTCATCAATGG-3′ and the reverse primer 5′-G GCAGTGATGGCATGGACTG-3′ for GAPDH. The corresponding PCR products were 1267 bp for TES and 358 bp for GAPDH.

### Protein extraction and western blotting

Forty-eight h after adenoviral transfection, protein expression was examined by Western blotting. GC cells (MKN45, SGC7901, MGC803, AGS, and HGC27) were lysed in RIPA lysis buffer (Beyotime, Shanghai, China), and lysates were harvested by centrifugation (13,000×*g* at 4 °C for 30 min. Western blotting was carried out as we previously described [3], using GAPDH as an internal control. The following primary antibodies and secondary antibodies were used:

A mouse monoclonal antibody against TES (1:500 dilution; Santa Cruz, Dallas, TX, USA), a rabbit monoclonal antibody against Mena (1:1000 dilution; Cell Signaling Technology, Boston, MA, USA), a rabbit polyclonal antibody against Lpd (1:1000 dilution; Sigma, St.Louis, MI, USA), HRP-conjugated rabbit anti-mouse IgG antibody (1:2000 dilution; Santa Cruz) and HRP-conjugated goat anti-rabbit IgG antibody (1:2000 dilution; Epitomics, Burlingame, CA, USA), a HRP-conjugated mouse anti-human GAPDH monoclonal antibody (1:5000 dilution; Shanghai Kangchen, Shanghai, China).

### Proliferation assay

MKN45 or SGC7901 cells were seeded in RPMI-1640 medium supplemented with 10% FBS in 96-well plates (500 cells per well). CellTiter 96^®^ AQueous One Solution Cell Proliferation Assay (Promega, Beijing, China) was used to assess cell viability according to the manufacturer’s instruction for 5 consecutive days. Triplicate independent experiments were performed. The proliferation rate was calculated as follows: proliferation rate = (OD_*n*_− OD_1_)/OD_1_ × 100% (*n*: days after transfection).

### Colony formation assay

MKN45 or SGC7901 cells were seeded onto 6-well plates (500 cells per well). After cultured in RPMI-1640 medium supplemented with 10% FBS for 12 days, surviving colonies (> 50 cells per colony) were counted after 0.5% (m/v) crystal violet staining. Colony-forming efficiency (CFE %) was defined as the ratio of the number of colonies formed to the number of cells inoculated. Triplicate independent experiments were performed.

### Cell cycle analysis

Forty-eight h after adenoviral transfection, GC cells were collected, washed twice with precooled phosphate buffer sodium (PBS) and fixed with ice-cold 75% ethanol at − 20 °C for 1 h. After washing with PBS, cells were resuspended in ice-cold PBS (500 µL) containing RNAase (100 µg) and incubated at 37 °C for 30 min, followed by propidium iodide (PI) staining at 4 °C for 30–60 min. Cell cycles of MKN45 and SGC7901 cells were analyzed on a FC500 flow cytometer (Beckman, Brea, CA, USA) using the Cylchred software (University Wales College Medicine, Cardiff, UK).

### Cell invasion and migration assay

Cell invasion and migration assay were performed using MKN45 and SGC7901 cells transfected with Ad-TES or Ad-Control. The invasion assay was performed in a Transwell comprising a polycarbonate membrane with 8-μm pores (Corning, Shanghai, China) placed in a 24-well plate. The Transwell insert was coated with 50 µL Matrigel Basement Membrane Matrix (BD Biosciences, Bedford, MA, USA). Cells in 100 μL of RPMI-1640 medium without FBS were added to the Transwell insert, and 0.5 mL of RPMI-1640 medium containing 20% FBS was placed in the lower chamber. The cells were incubated at 37 °C and allowed to invade through the Matrigel layer. After 48 h, cells on the lower surface of the Transwell insert were fixed with 75% methanol and stained with 0.5% (m/v) crystal violet. The stained cells were counted in 10 random fields with 200× magnification. The migration assay was similar to the invasion assay, except that the Transwell insert was uncoated. Each experiment was performed in triplicate.

### Apoptosis assay

MKN45 and SGC7901 cells were washed twice with ice-cold PBS and resuspended in 400 µL 1× Binding Buffer (Bestbio, Shanghai, China), according to the manufacturer’s protocol. After incubation with 5 µL Annexin V-FITC (Bestbio) for 15 min at room temperature in the dark, 10 µL PI (Bestbio) was added, and the cells stained with PI and Annexin V were counted using flow cytometer (Beckman).

### Small interfering RNA (siRNA) transfection

siRNA targeting Mena (siMena, synthesized by GenePharma Company, Shanghai, China) were used to knockdown Mena expression in MKN45 and SGC7901 cells. The cells were seeded in 6-well plates (1 × 10^6^ cells per well). When an approximately 50% cell confluence was observed, the cells were transfected with 400 pmol of siMena or siNC (negative control) at 37 °C for 48 h using Lipofectamine RNAi MAX reagent (Invitrogen), according to the manufacturer’s instructions. The expression level of Mena was examined 48 h after the transfection. siRNA sequences used in this study were as follows:

Sense sequence 5′-GGUCCUAUGAUUCAUUACATT-3′ and antisense sequence 5′-UGUAAUGAAUCAUAGGACCTT-3′ for siMena 1;

Sense sequence: 5′-GCGAGAAAGAAUGGAAAGATT-3′ and antisense sequence 5′-UCUUUCCAUUCUUUCUCGCTT-3′ for siMena 2;

Sense sequence: 5′-UUCUCCGAACGUGUCACGUTT-3′ and antisense sequence 5′-ACGUGACACGUUCGGAGAATT-3′ for siNC.

### Immunoprecipitation-based mass spectrometry (IP-based MS)

A Pierce^®^ Classic IP Kit (Thermo Scientific) was used to perform the protein interaction assay, according to the manufacturer’s protocol. In brief, after lysing in IP Lysis/Wash Buffer (0.025 mol/L Tris, 0.15 mol/L NaCl, 0.001 mol/L EDTA, 1% NP-40, and 1% protease inhibitor cocktail) at 4 °C for 5 min, cell lysates were collected by centrifugation (13,000×*g*) at 4 °C for 10 min. The supernatants were transferred to new tubes immediately. Total proteins were quantified with BCA Protein Quantification Kit (Beyotime) and adjusted to 3.5 μg/μL with PBS. An amout of 10 μg primary antibody against the target protein were added and incubated at 4 °C overnight. The mixtures were then incubated with 20 μL Protein A/G Plus Agarose for 6 h at 4 °C. After incubation, the Protein A/G Plus Agarose were washed three times with IP Lysis/Wash Buffer. Collected precipitates were eluted with 50 μL elution buffer at 100 °C for 10 min. Eluted proteins were separated by sodium dodecyl sulfate polyacrylamide gel electrophoresis (SDS-PAGE) and the protein bands were stained with silver solution (1×; Beyotime). The bands of interest were analyzed using MS (Institutes of Life and Health Engineering, Jinan University, Guangzhou, Guangdong, China). The antibodies used in immunoprecipitation were as follows: a mouse monoclonal antibody against TES (1:100 dilution; Sigma) and a rabbit monoclonal antibody against Mena (1:50 dilution; Cell Signaling Technology).

### Tumorigenicity assays in nude mice

Five-week-old BALB/c nude mice (Guangdong Medical Experimental Animal Center, Guangzhou, Guangdong, China) were randomly assigned to four groups (7 mice per group) before inoculation. At 48 h after adenoviral transfection, MKN45 and SGC7901 cells were injected subcutaneously into the groin of mice respectively (5 × 10^6^ cells suspended in 200 μL PBS per mouse). The length (L) and width (W) of the tumor was measured with calipers every 3 days. The tumor volume was calculated as (L × W^2^)/2. Thirty-two days after tumor inoculation, the mice were sacrificed by cervical dislocation, and tumor weight was assessed.

### Metastasis assay in nude mice

To investigate the suppressive effect of TES on tumor metastasis, 1 × 10^6^ cells (MKN45 cells transfected with Ad-TES or Ad-Control) in 200 μL PBS were injected intravenously through the lateral tail vein into 5-week-old nude mice (8 mice per group). After 6 weeks, the mice were necropsied after anesthesia. Their lungs were fixed in 3.7% formaldehyde, 5% glacial acetic acid, and 72% ethanol for 24 h before proceeding to paraffin embedding. Serial 5-μm sections were stained with hematoxylin and eosin (H&E) for histopathological examination. Metastasis lesions from 10 random high-power fields were counted. All animal experiments were conducted in compliance with the guidelines of the laboratory animal ethics committee of Sun Yat-sen University (Guangzhou, Guangdong, China).

### Immunohistochemistry (IHC) and semi-quantitative analysis

IHC was performed on GC tissue sections as previously described [3]. The antibodies used in IHC were as follows: a mouse monoclonal antibody against TES (1:300 dilution; Santa Cruz) and a rabbit monoclonal antibody against Mena (1:500 dilution, Cell Signaling Technology). The intensity and extent of immunostaining were evaluated for all tumor samples by three pathologists under double-blinded conditions. In brief, the percentage of positive staining was scored as 0 (0–9%), 1 (10–25%), 2 (26–50%), or 3 (51–100%), and the intensity as 0 (no staining), 1 (weak staining), 2 (moderate staining) or 3 (dark staining). The total immunostaining score was calculated as the product of extent and intensity, ranging from 0 to 9.

Based on IHC scores, expression levels of Mena and TES were defined as low (score 0–3) or high (score 4–9) in subgroups.

### Statistical analysis

Student *t* test was performed for comparison of continuous variables between groups. Repeated measurement analysis of variance (ANOVA) analysis was used to compare curves of tumor growth. Chi square test was used to examine differences of categorical variables between subgroups. Kaplan–Meier survival curve was plotted and compared by log-rank test. The patients did not have an event during the observation time were described as censored. Multivariate Cox proportional hazards regression model was used to calculate the hazard ratio (HR) and 95% confidence interval (CI) for prognosis evaluation. All statistical analyses were performed using the SPSS Statistics 22.0 software (IBM, Armonk, NY, United States), and *P* < 0.05 was considered statistically significant.

## Results

### TES inhibits GC cell proliferation in vitro

**Fig. 1:**
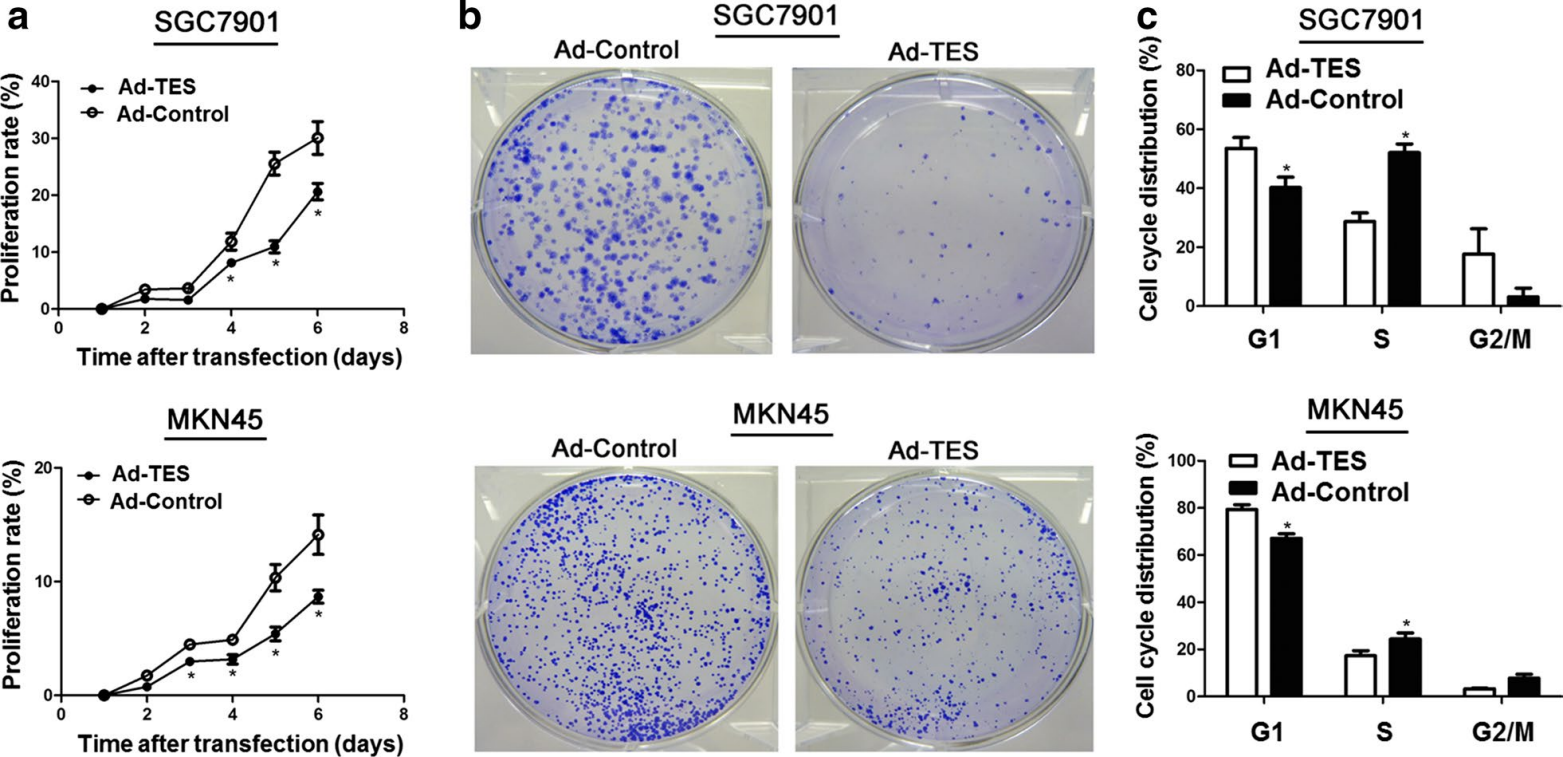
Overexpression of testin LIM domain protein (TES) suppresses gastric cancer (GC) cell viability in vitro. **a** Proliferation rate of Ad-TES transfected SGC7901 and MKN45 cells were compared with Ad-Control cells by proliferation assay. The results are expressed as mean ± standard deviation (SD) of at least three independent experiments. Student’s *t*-test, *P < 0.05. **b** Representative pictures of colony formation assay in Ad-TES transfected SGC7901 and MKN45 cells. The cells transfected with Ad-Control were used as control. **c** Summary of cell cycle distributions detected by flow cytometry shows that the proportion of cells at S phase was much lower in Ad-TES-transfected SGC7901 and MKN45 cells than that in control cells. Values are expressed as mean ± SD of at least three independent experiments. Student’s *t* test, *P < 0.05

To evaluate the function of TES in GC cells, the SGC7901 and MKN45 cells with low TES expression were transfected with Ad-TES and Ad-Control. The transfection efficiency of Ad-TES in SGC7901 and MKN45cells were 81.9% and 98.0%, respectively (Additional file 1: Figure S1). The RT-PCR and Western blotting results confirmed that the mRNA and protein levels of TES in the cells transfected with Ad-TES were markedly higher than those in the cells transfected with Ad-Control (Additional file 1: Figure S2). As shown in Fig. 1a, the proliferation rates of both SGC7901 and MKN45 cells were significantly suppressed after Ad-TES transfection. Similarly, colony formation assay revealed that Ad-TES transfection significantly inhibited colony formation (Fig. 1b). To further investigate the suppressive effect of TES on GC cell growth, we determined cell cycle distributions of Ad-TES and Ad-Control transfectants using flow cytometry based on the DNA content. As shown in Fig. 1c, TES overexpression significantly increased the proportion of cells at G_1_ phase and decreased the proportion of cells at S phase, suggesting that TES inhibited cell cycle proceeding in GC cells. However, TES overexpression did not observably influence the apoptosis of GC cells (Additional file 1: Figure S3).

### TES suppresses carcinogenesis of GC cells in vivo

**Fig. 2:**
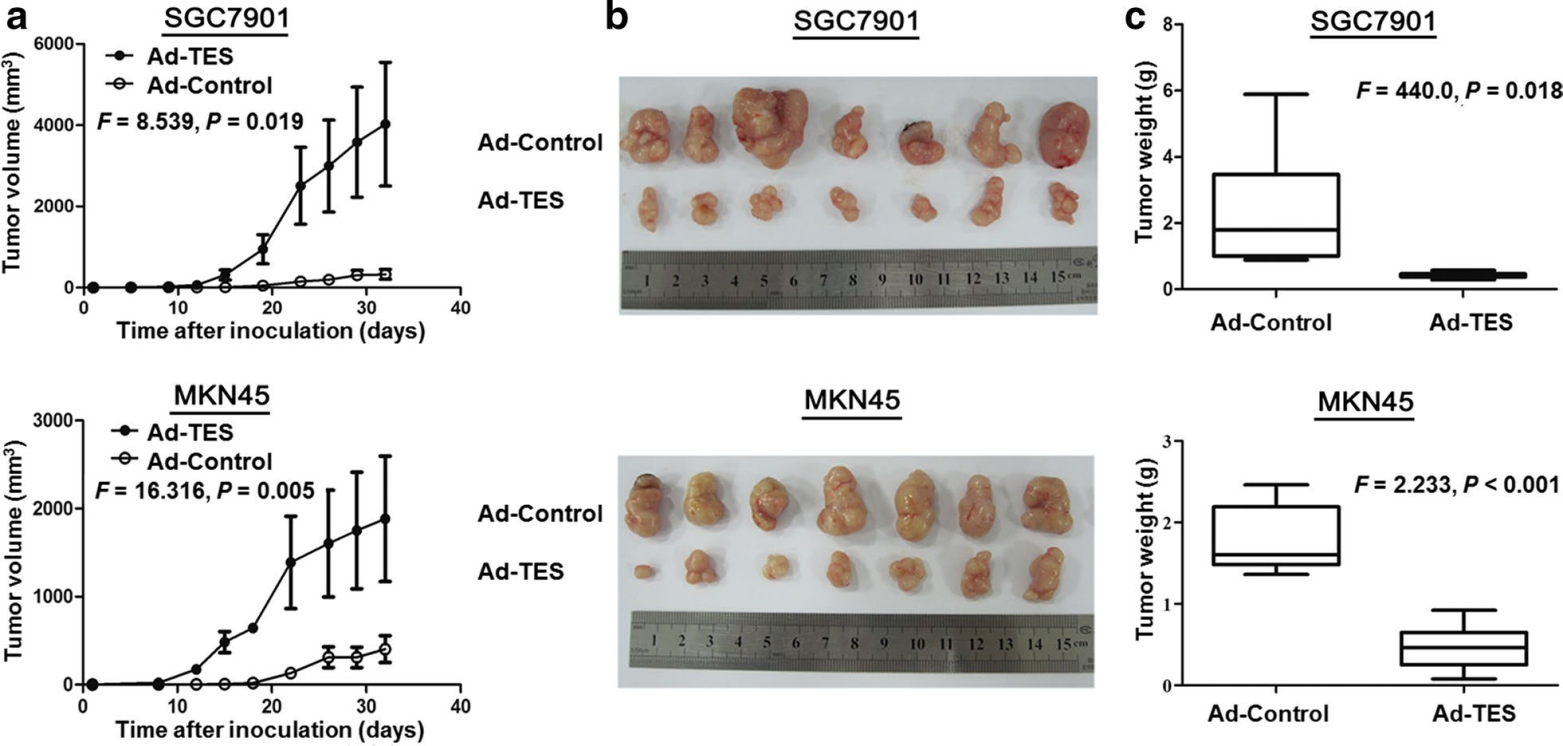
Overexpression of TES suppresses the tumorigenicity of SGC7901 and MKN45 cells in vivo. **a** Tumor growth curves of Ad-TES-transfected SGC7901 and MKN45 cells in nude mice were compared with Ad-Control-transfected cells in tumorigenicity assay. The data were analyzed by ANOVA. The average tumor volume is expressed as mean ± SD in seven inoculated nude mice for each group. **b** Representative pictures of dissected tumors from nude mice 6 weeks following injection of Ad-TES or Ad-Control-transfected SGC7901 and MKN45 cells and control cells (*n* = 7 mice per group), respectively. The tumor volumes are smaller in the Ad-TES group than in the Ad-Control group. **c** Tumor weights were compared between the Ad-TES group and the Ad-Control group by ANOVA. The results are expressed as mean ± SD

We next analyzed the effects of TES on GC carcinogenesis in vivo. As shown in Fig. 2a, Ad-TES transfection remarkably delayed the tumor formation of SGC7901 and MKN45 cells in nude mice as compared with Ad-Control transfection. The mean tumor volume of the Ad-TES group was significantly smaller than that of the Ad-Control group (329.88 mm^3^ vs. 4026.26 mm^3^ for SGC7901, *P* = 0.018; 404.22 mm^3^ vs. 1884.46 mm^3^ for MKN45, *P* < 0.001; Fig. 2b) at the end of the experiment. Similarly, the mean tumor weight of the Ad-TES group was markedly lower than that of the Ad-Control group (0.42 g vs. 2.30 g for SGC7901, *P* = 0.018; 0.46 g vs. 1.79 g for MKN45, *P* < 0.001; Fig. 2c).

### TES inhibits migration and invasion of GC cells

**Fig. 3:**
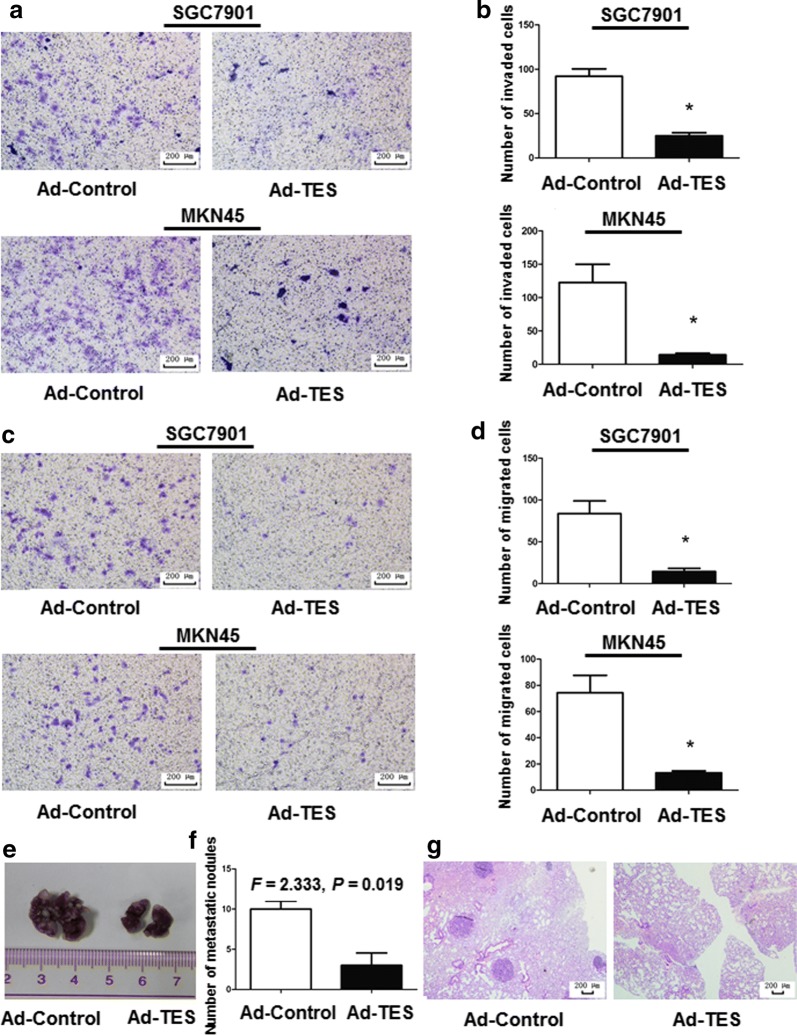
Overexpression of TES suppresses migration and invasion of GC cells both in vitro and in vivo. **a** Representative pictures show that the Ad-TES- and Ad-Control-transfected SGC7901 and MKN45 cells invaded through the matrigel. Magnification: ×100. **b** Invaded, Ad-TES-transfected SGC7901 and MKN45 cells were quantified. Ad-Control-transfected SGC7901 and MKN45 cells were used as controls. The results are expressed as mean ± SD of at least three independent experiments. Student’s *t*-test, *P < 0.05. **c** Representative pictures show that the Ad-TES- and Ad-Control-transfected SGC7901 and MKN45 cells migrated through the uncoated transwell membrane. Magnification: ×100. **d** Migrated Ad-TES transfected SGC7901 and MKN45 cells were quantified. Ad-Control-transfected SGC7901 and MKN45 cells were used as controls. The results are expressed as mean ± SD of at least three independent experiments. Student’s *t*-test, *P < 0.05. **e** Representative lungs derived from nude mice inoculated with Ad-TES- or Ad-Control-transfected MKN45 cells are shown. The formation of metastatic nodules at the surface of lungs could be significantly suppressed by TES overexpression. **f** Metastatic nodules in the lung were quantified 6 weeks after tail vein injection of Ad-TES- and Ad-Control-transfected cells (8 mice per group). The nodules were counted in 10 randomly selected high-power fields under a microscope. The results are expressed as mean ± SD. **g** Representative hematoxylin and eosin staining pictures of the lung sections from mice inoculated with Ad-TES- and Ad-Control-transfected MKN45 cells. Magnification: ×40

The invasion assays revealed that the invasion ability of SGC7901 and MKN45 cells overexpressing TES were significantly decreased as compare with their control counterparts (Fig. 3a, b). Similarly, significantly fewer SGC7901 and MKN45 cells transfected with Ad-TES migrated into the lower compartment of the migration chamber than those transfected with Ad-Control (Fig. 3c, d). Thus, these results suggest that TES overexpression can inhibit GC cell migration and invasion.

### TES inhibits pulmonary metastasis in vivo

To determine the impact of TES on GC metastasis, nude mice were injected with MKN45 and SGC7901 cells transfected with Ad-TES, whereas the cells transfected with Ad-Control were used as controls. Six weeks after injection, the body weights of the mice did not differ between the Ad-TES group and the Ad-Control group. However, there were fewer metastatic nodules on the surface of excised lungs in the Ad-TES group than in the Ad-Control group (Fig. 3e). Lung sections stained with H&E showed that the Ad-TES group had significantly fewer lung metastasis lesions than the Ad-Control group (10 vs. 3, *P* = 0.019; Fig. 3f, g), indicating a suppressive effect of TES on GC metastasis.

### TES interacts with Mena

**Fig. 4:**
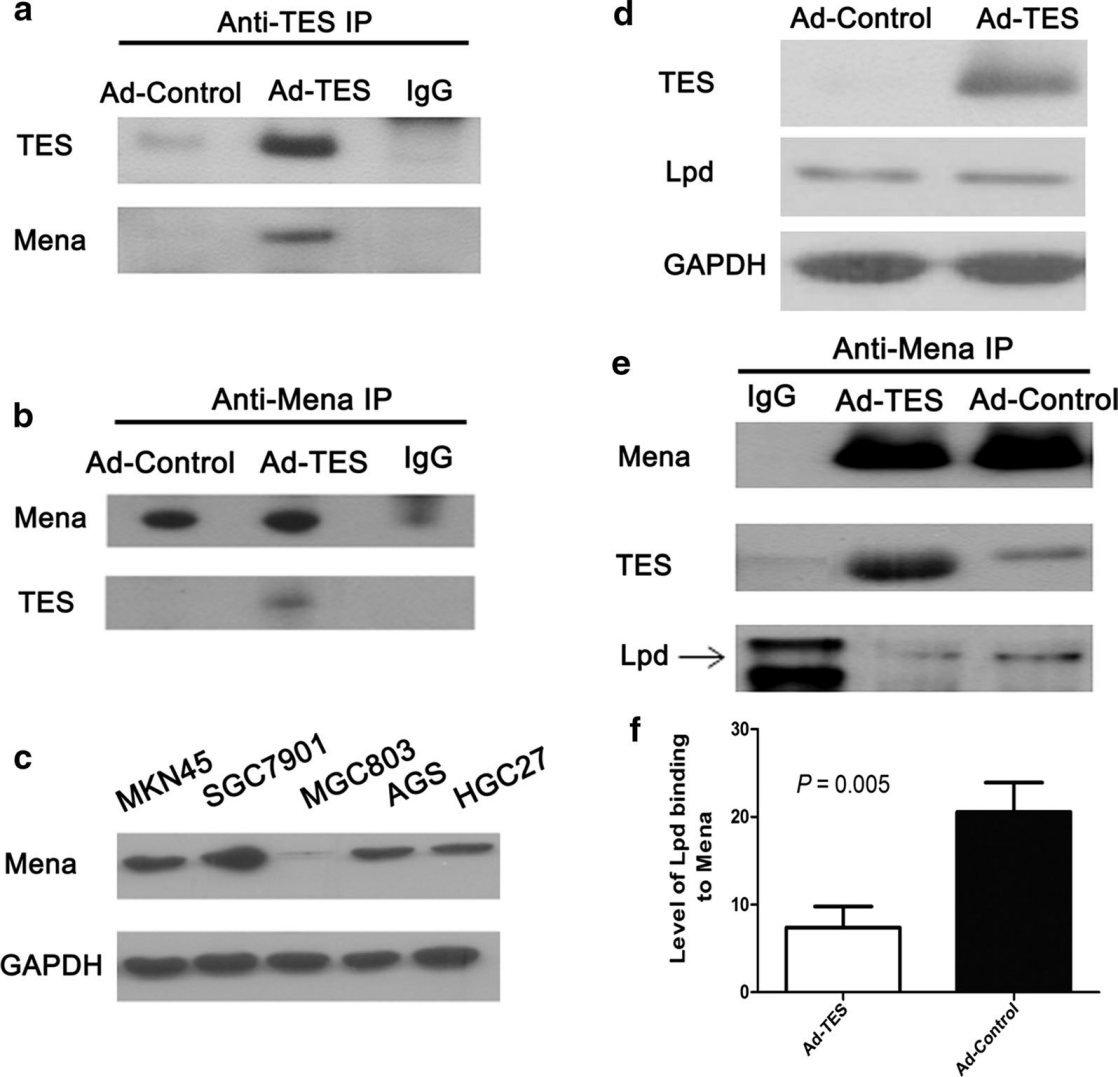
TES inhibits the interaction between Mena and Lpd. **a** Western blotting of immunoprecipitates prepared using antibodies against TES revealed that TES interacted with Mena. **b** Western blotting of immunoprecipitates prepared using antibodies against Mena revealed that Mena interacted with TES. **c** Mena protein expression in GC cell lines detected by Western blotting. **d** The association of Lpd and TES expression in Ad-TES- or Ad-Control-transfected MKN45 cells detected by Western blotting. Overexpression of TES had no effect on the Lpd expression in MKN45 cells. **e** Western blotting of immunoprecipitates from Ad-TES-transfected MKN45 cells show that overexpression of TES reduced the level of Lpd in the immunoprecipitates prepared using antibodies against Mena. The immunoprecipitates from Ad-Control-transfected MKN45 cells were served as control. The loading volume of immunoprecipitates was 10 μL for each sample. Three independent experiments were performed. **f** The level of Lpd (band intensity) binding to Mena in Ad-TES-transfected cells and Ad-Control-transfected cells were compared by Student’s *t*-test. The results are expressed as mean ± SD of at least three independent experiments

To further investigate the potential molecular mechanism of TES in cell cycle arrest, cell migration and invasion, we performed IP-based MS study to identify TES-interacting proteins. Among the top listed protein candidates that were identified as putative binding partners of TES (Additional file 1: Table S1), Mena, encoded by the enabled homolog gene (*ENAH*), was of primary interest. Western blotting of the immunoprecipitates confirmed the interaction between TES and Mena (Fig. 4a, b).

### TES inhibits the interaction between Mena and Lpd

Previous studies have implicated the interaction between Mena and Lpd [15]. We therefore explored the impact of TES on the interaction between Mena and Lpd. The protein expression of Mena was detected in GC cell lines (Fig. 4c). MKN45 cells with high expression of Mena were chosen for the subsequent experiments. Overexpression of TES had no effect on Lpd expression in MKN45 cells (Fig. 4d). However, our immunoprecipitation assay revealed that TES overexpression significantly reduced the binding of Lpd to Mena (*P* = 0.005; Fig. 4e, f), indicating that TES may inhibit the interaction between Lpd and Mena.

### TES inhibits migration and invasion of GC cells in a Mena-dependent fashion

**Fig. 5:**
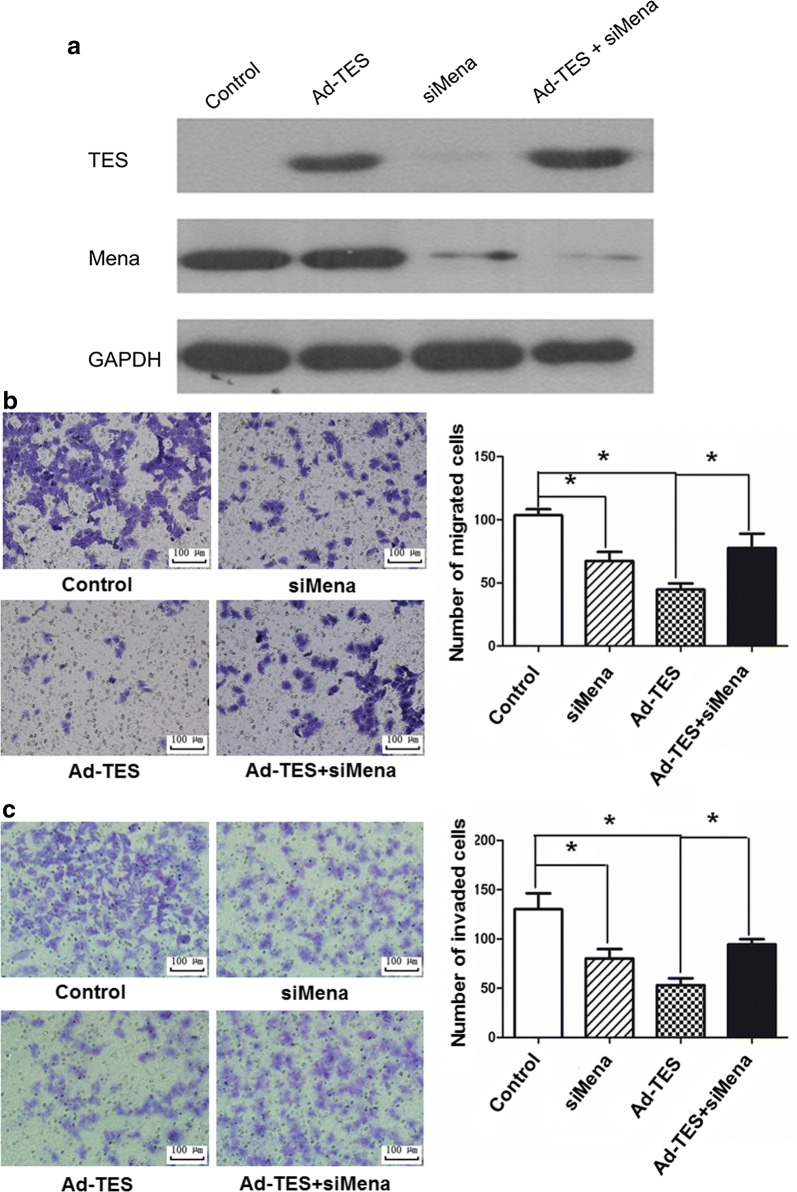
TES inhibits migration and invasion of GC cells in a Mena-dependent fashion. **a** Expression of TES and Mena in siMena-transfected MKN45 cells was detected by Western blotting. Overexpression of TES significantly suppressed the migration (**b**) and invasion (**c**) of siNC-transfected MKN45 cells compared with those of siMena-transfected MKN45 cells. Control: MKN45 cells transfected with Ad-Control and siNC. siMena: MKN45 cells transfected with Ad-Control and siMena. Ad-TES: MKN45 cells transfected with Ad-TES and siNC. Ad-TES + siMena: MKN45 cells transfected with Ad-TES and siMena. The results are expressed as the mean ± SD of three independent experiments. Magnification: ×200. *P < 0.05

To test whether down-regulation of Mena might function in TES-induced inhibition of GC cell migration and invasion, we inhibited Mena expression with siRNA in Ad-TES transfected cells (Fig. 5a). The results of migration and invasion assays revealed that Mena silencing significantly promoted migration and invasion of Ad-TES-transfected cells (Fig. 5b, c), suggesting that TES inhibits migration and invasion of GC cells in a Mena-dependent fashion.

### TES expression is associated with OS of GC patients in a Mena-dependent fashion

**Fig. 6:**
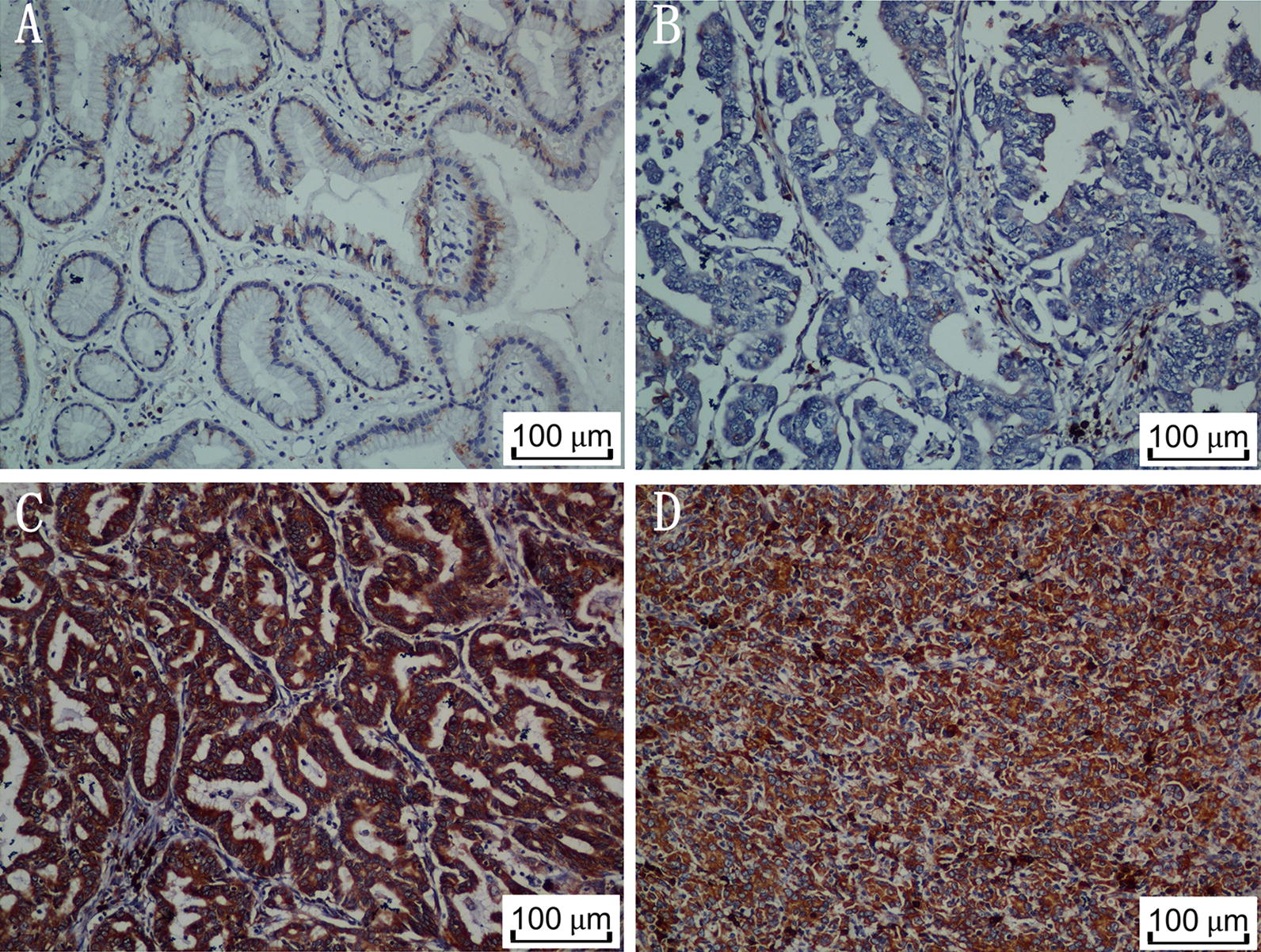
Mena protein expression in GC specimens was detected by immunohistochemistry. Weak Mena staining was observed in noncancerous gastric mucosa (**A**) and in well-differentiated gastric adenocarcinoma (**B**). Strong Mena staining was observed in moderately differentiated (**C**) and poorly differentiated gastric adenocarcinoma (**D**). Magnification: ×200

**Fig. 7:**
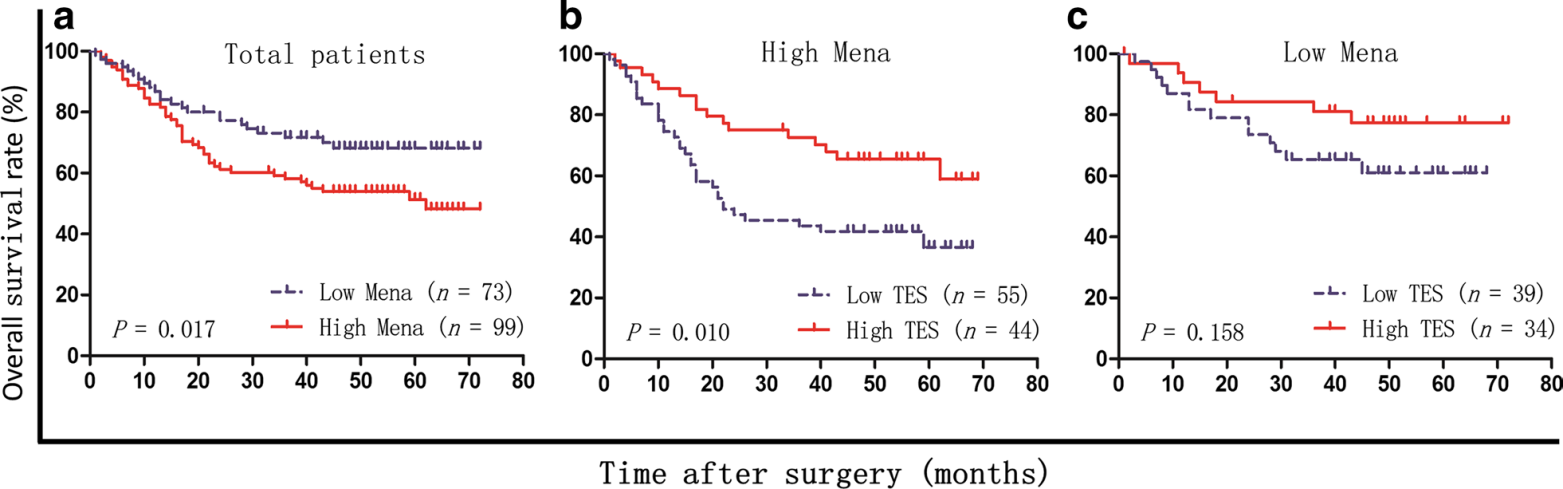
Kaplan–Meier overall survival curves of 172 GC patients after gastrectomy. **a** The survival rate of patients with high Mena expression was significantly lower than that of patients with low Mena expression (log-rank test, *P* = 0.017). **b** Among the 99 patients with high Mena expression, the survival rate of patients with high TES expression was significantly higher than that of patients with low TES expression (log-rank test, *P *= 0.010). **c** Among the 73 patients with low Mena expression, there was no significant association between TES expression and patient survival (log-rank test, *P* = 0.158)

Since TES inhibited the migration and invasion of GC cells in a Mena-dependent manner, we investigated the impact of Mena on the association between TES expression and the prognosis of 172 GC patients. Representative images of IHC staining using antibodies against Mena are shown in Fig. 6. Similar to previous findings [16], high Mena expression was significantly associated with high rate of lymph node metastasis (*P* = 0.024, Table 1) and worse prognosis (*P* = 0.017, Fig. 7a). We next analyzed the association of TES with clinicopathological characteristics and OS of patients in different subgroups divided by Mena expression. Interestingly, in the group with high Mena expression, TES expression was negatively associated with tumor infiltration (*P* = 0.005), local lymph node metastasis (*P* = 0.003), and TNM stage (*P* = 0.003) (Additional file 1: Figure S4, Table 2). Moreover, high TES expression was significantly associated with long OS of patients with high Mena expression (*P* = 0.010; Fig. 7b). However, in the group with low Mena expression, there was no significant association between TES expression and clinicopathological characteristics (Table 2) or prognosis (*P* = 0.158, Fig. 7c).

**Table 1:** Association between Mena expression and clinicopathological characteristics of 172 gastric cancer patients

Characteristic	Total (cases)	Mena expression (cases)		χ^2^	*P* value
		High	Low		
*All*	172	99	73		
*Age (years)*				0.401	0.527
< 55	80	44	36		
≥ 55	92	55	37		
*Gender*				0.351	0.553
Male	115	68	47		
Female	57	31	26		
*Tumor infiltration*				13.786	0.008
T1	27	10	17		
T2	24	9	15		
T3	3	1	2		
T4a	82	56	26		
T4b	36	23	13		
*Local lymph node metastasis*				5.115	0.024
N0	59	27	32		
N1–N3	113	72	41		
*Distant metastasis*				0.061	0.806
M0	152	88	64		
M1	20	11	9		
*TNM stage*				16.565	0.001
0–I	30	9	21		
II	69	40	29		
III	50	38	12		
IV	23	12	11

**Table 2:** Association between TES expression and clinicopathological characteristics of gastric cancer patients with high or low Mena expression

Characteristic	High Mena expression group (cases)					Low Mena expression group (cases)				
	Total (cases)	High TES expression	Low TES expression	χ^2^	*P* value	Total (cases)	High TES expression	Low TES expression	χ^2^	*P* value
*Tumor infiltration*				8.141	0.005				1.486	0.854
T1–T2	19	14	5			32	16	16		
T3–T4	80	30	50			41	18	23		
*Local lymph node metastasis*				10.106	0.003				0.269	0.643
N0	27	19	8			32	16	16		
N1–N3	72	25	47			41	18	23		
*Distant metastasis*				3.457	0.105				0.333	0.725
M0	88	42	46			64	29	35		
M1	11	2	9			9	5	4		
*TNM stage*				13.109	0.003				2.560	0.489
0–I	9	7	2			21	9	12		
II	40	23	17			29	13	16		
III	38	12	26			12	8	4		
IV	12	2	10			11	4	7		

## Discussion

TES has been identified as a TSG in many types of tumors, such as uterine cancer [7], ovarian carcinoma [17], breast cancer [18], endometrial carcinoma [19], head and neck squamous cell carcinoma [20], and non-small cell lung cancer [21]. In the present study, we investigated the effect of TES on the viability of GC cells both in vitro and in vivo. Our results showed that exogenous expression of TES in GC cells suppressed cell proliferation and colony formation in vitro as well as tumorigenicity in vivo. This suppressive effect is caused by inducing cell cycle arrest. These findings suggest an important tumor suppressive role for TES in GC carcinogenesis.

In the present study, we also found that exogenous expression of TES significantly inhibited the migration and invasion of GC cells in vitro. Furthermore, we demonstrated that TES suppressed the formation of metastatic lesions in the lungs of nude mice. These data indicate the potential role of TES in inhibition of GC invasion and metastasis. Consistent with our findings, a previous study showed that overexpression of TES significantly inhibited breast cancer cell invasion and reduced breast cancer cell metastasis to the lung through blocking the secretion of matrix metallopeptidase-2 (MMP-2) [18]. Overexpression of TES also markedly inhibited the invasion and metastasis of endometrial carcinoma [19] and non-small cell lung cancer [21]. Thus, TES down-regulation may play important roles in the progression of different types of human cancers.

To explore the potential mechanisms underlying the tumor suppressive roles of TES in GC, we analyzed the potential proteins that interact with TES using IP-based MS. Mena, a member of the Ena/VASP family, was identified to be an interacting partner of TES. Previous studies demonstrated that Mena regulated cell motility by promoting actin polymerization at the leading edge of migrating cells [5, 12]. It is frequently upregulated in several human cancers, particularly in invasive tumor cells [12, 22]. Deficiency of Mena could reduce tumor cell invasion and intravasation in mice [23], and elevated Mena expression was associated with increased invasiveness of breast tumors and enhanced cancer metastasis to the lungs in severe combined immune-deficient mice [12, 24]. In line with these findings, we found that Mena silencing significantly inhibited the invasion of GC cells. Moreover, Mena silencing attenuated the invasion-suppressive effects of TES on GC cells. These data collectively suggest that TES may inhibit GC cell migration and invasion partly through its interaction with Mena.

Ena/VASP family proteins can recruit MRL proteins to the leading edge of filopodia and lamellipodia to regulate cell lamellipodial spreading and motility [5, 24]. Previous studies have demonstrated that Lpd promoted invasive 3D cancer cell migration via its interactions with Ena/VASP proteins [25]. Additionally, upregulation of Lpd is associated with poor prognosis in breast cancer patients [25]. In the present study, we found that TES inhibited the interaction between Mena and Lpd, which is supported by the previous findings that TES could bind to Ena/VASP in competition with Lpd [5]. These data suggest that TES may inhibit the migration and invasion of GC cells by suppressing the interaction between Mena and Lpd, which may inhibit lamellipodial protrusion and cell motility as well as the subsequent intracellular signaling pathways [26].

Previous studies reported that TES localizes to the regions of cell–cell and cell–substratum contact, and affects cell spreading and protrusions, suggesting that TES has a role in cell adherence and cell motility. Besides, TES inhibits the invasion and angiogenesis of breast cancer partially through miR-29b-mediated MMP-2 inhibition [18]. TES functions as a necessary tumor suppressor of colorectal cancer progression by activating mitogen-activated protein kinase (p38-MAPK) signaling pathways [27]. TES suppressed the epithelial-mesenchymal transition in endometrial cancer [28] and breast cancer [29]. Other investigators have reported that TES binds to the EVH1 domain of Mena with its LIM3 domain [5]. We observed that TES inhibited the interaction between Mena and Lpd, suggesting that the molecular mechanism of TES in GC metastasis is associated with cell spreading and pseudopodium protrusion. These results enrich our understanding of the molecular mechanisms of GC metastasis. However, the mechanism by which TES regulates cell spreading and cell pseudopodium protrusion requires further investigation.

We also investigated the association between TES and Mena expression in 172 GC patients. In the patients with high Mena expression, TES expression was negatively associated with tumor infiltration, lymph node metastasis, TNM stage, and prognosis. However, in the patients with low Mena expression, there was no significant association between TES expression and clinicopathological parameters or prognosis. These results suggested that TES expression is associated with GC prognosis and pathological parameters in a Mena-dependent fashion. Analysis of these results indicated that Mena may be a potential inhibitory target in GC metastasis.

## Conclusions

In conclusion, our study demonstrated that TES suppressed GC cell proliferation and colony formation, induced cell cycle arrest in vitro, and suppressed tumorigenicity in vivo. Furthermore, we reported that TES inhibited GC cell migration and invasion in a Mena-dependent fashion. The TES-mediated suppression of migration and invasion of GC cells depended on its interaction with Mena, which inhibited the interaction between Lpd and Mena. Thus, these findings provide a better understanding of the development and progression of GC and indicate that TES may be used as a potential prognostic marker and therapeutic target for GC patients.

## Additional file

**Additional file 1.** Additional table and figures.

## Abbreviations

GC: gastric cancer
Lpd: lamellipodin
STR: short-tandem repeat analysis
FBS: fetal bovine serum
siRNA: small interfering RNA
Ad-TES: TES-recombinant adenoviral expression vector
Ad-Control: control vector
MOI: multiplicity of infection
CFE: colony-forming efficiency
H&E: hematoxylin and eosin
