# Supplementary material for: TES functions as a Mena-dependent tumor suppressor in gastric cancer carcinogenesis and metastasis
Source: Cancer Commun (Lond). 2019 Feb 6;39:3. doi: 10.1186/s40880-019-0347-y (PMC6366075; doi:10.1186/s40880-019-0347-y)

**Additional file tables**

**Table S1**. TES interaction partners identified using mass spectrometry.

| **Protein** | **Gene name** | **Number of peptides** | **Score^1^** |
| --- | --- | --- | --- |
| Actin, cytoplasmic 2 | ACTG1 | 35 | 640 |
| Isoform 1 of POTE ankyrin domain family member E | POTEE | 15 | 260 |
| Isoform A of Lamin-A/C | LMNA | 14 | 248 |
| Isoform 1 of Myosin-Ib | MYO1B | 11 | 222 |
| Integrin alpha-5 | ITGA5 | 5 | 141 |
| Isoform Beta-1C of Integrin beta-1 | ITGB1 | 6 | 105 |
| Isoform 3 of LIM and calponin homology domains-containing protein 1 | LIMCH1 | 3 | 92 |
| LIM domain 7 | LMO7 | 3 | 90 |
| Isoform 2 of Protein enabled homolog | **ENAH (MENA)** | 2 | 65 |
| cDNA FLJ61629, highly similar to Clathrin interactor 1 | CLINT1 | 1 | 64 |
| Isoform 2 of Zinc finger CCCH-type antiviral protein 1 | ZC3HAV1 | 2 | 63 |
| Actin, aortic smooth muscle | ACTA1 | 3 | 45 |
| Isoform Beta of LIM domain and actin-binding protein 1 | LIMA1 | 4 | 56 |
| Beta-actin-like protein 2 | ACTBL2 | 8 | 47 |

1. Profound protein score

**Additional figure legends**

**Figure S1.** Representative images of SGC7901 and MKN45 cells transfected with Ad-TES or Ad-Control. The transfection efficiency of Ad-TES in SGC7901and MKN45 cells was 81.9% and 98.0%, respectively. Magnification: 100×.

**
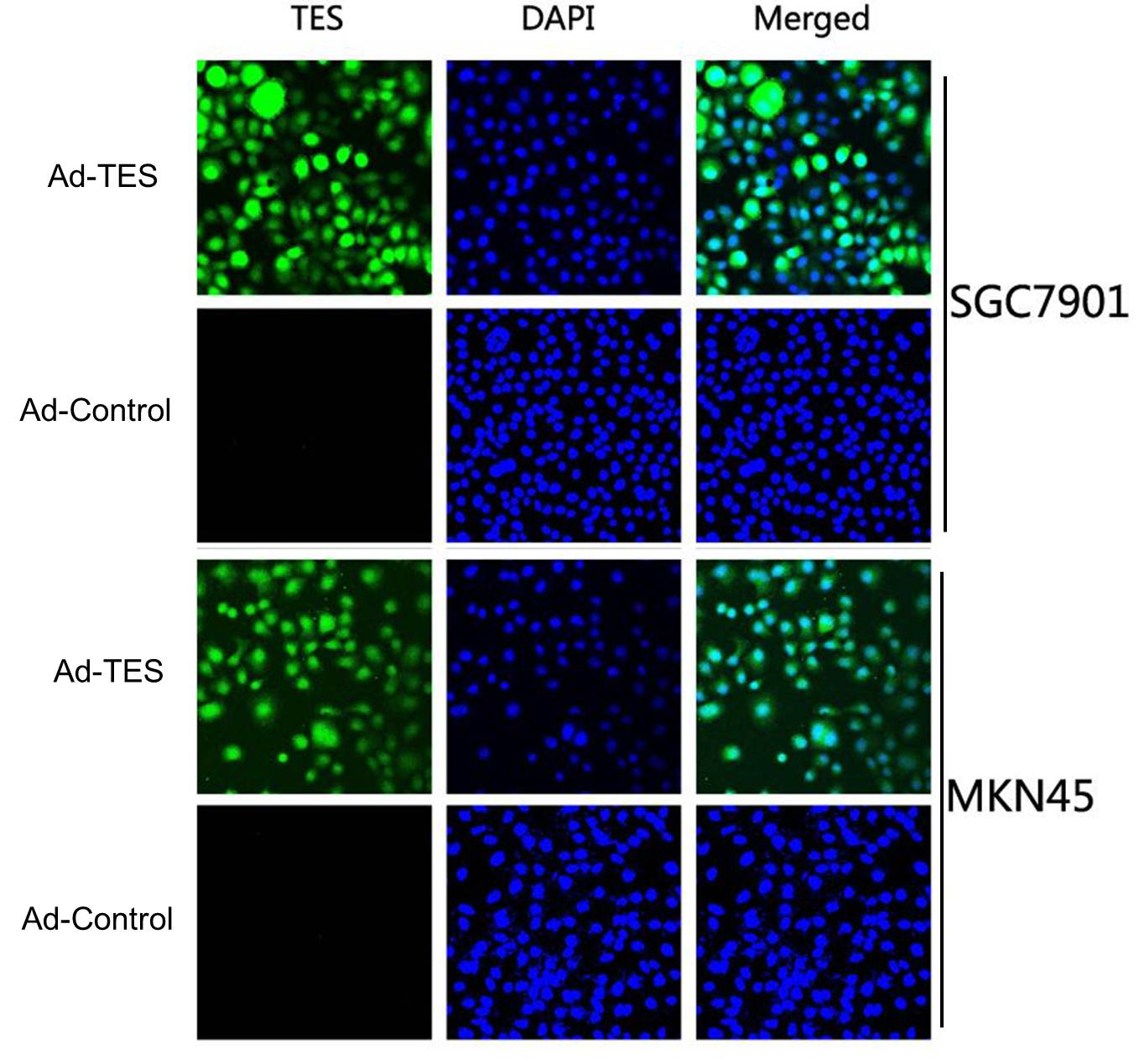
Figure S2.** The mRNA and protein expression of TES in Ad-TES- or Ad-Control-transfected SGC7901 and MKN45 cells was detected by RT-PCR (A) and Western blotting (B). The mRNA and protein levels of TES in Ad-TES-transfected GC cells were markedly higher than that in Ad-Control-transfected GC cells. MOI: multiplicity of infection.

**
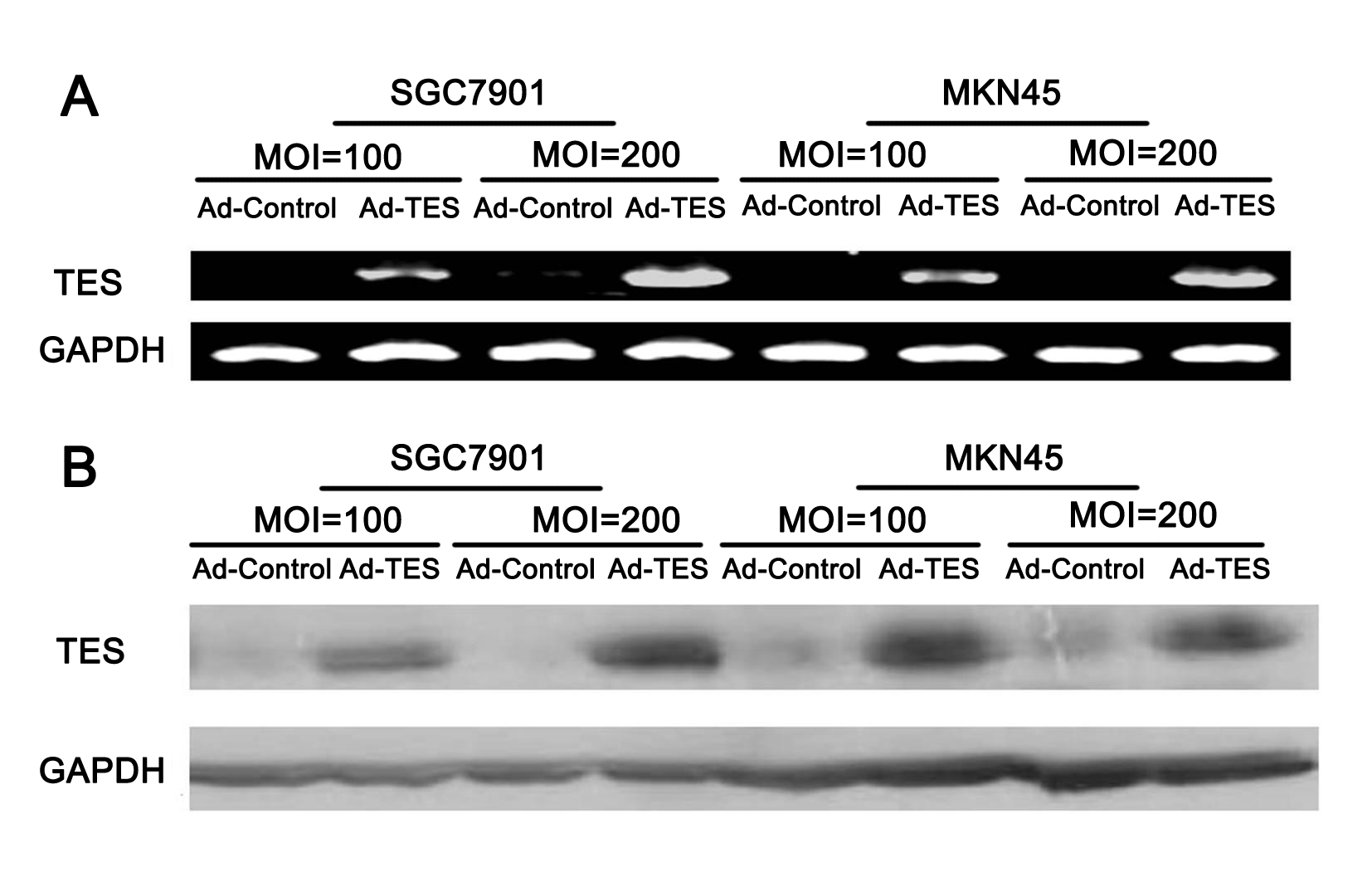
Figure S3.** Overexpression of TES has no significant effect on the apoptosis of GC cells. The apoptosis of SGC7901 (A) and MKN45 cells (B) at 3-7 days after Ad-TES transfection. The cells transfected with Ad-Control were used as control.

**
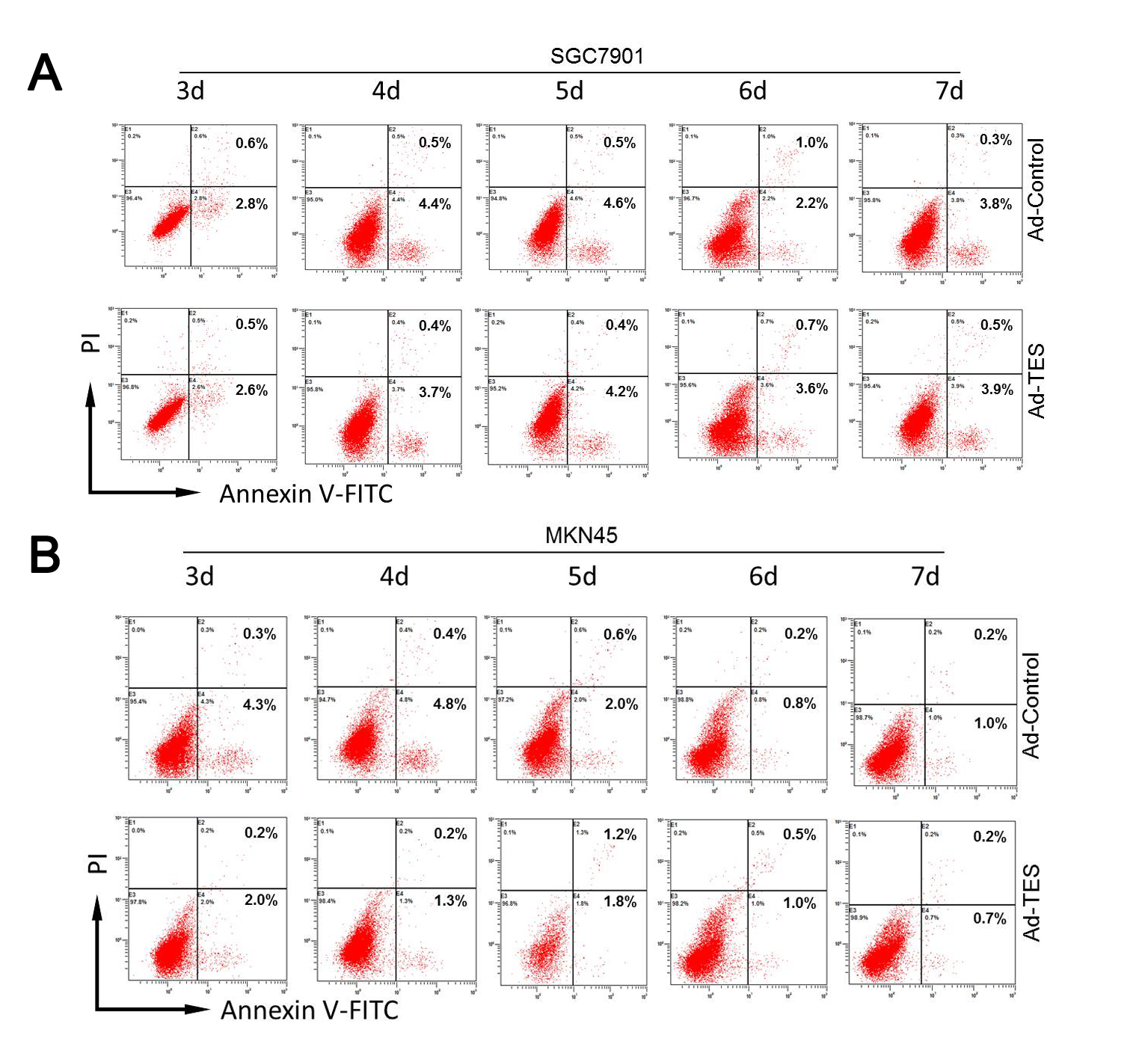
**

**Figure S4.** TES protein expression in GC specimens is detected by immunohistochemistry. (A) Strong TES staining is observed in noncancerous gastric mucosa. (B) Weak TES staining is observed in well-differentiated gastric cancer tissues. TES-negative staining is observed in moderately differentiated (C) and poorly differentiated gastric adenocarcinoma (D). Magnification: 200 ×.


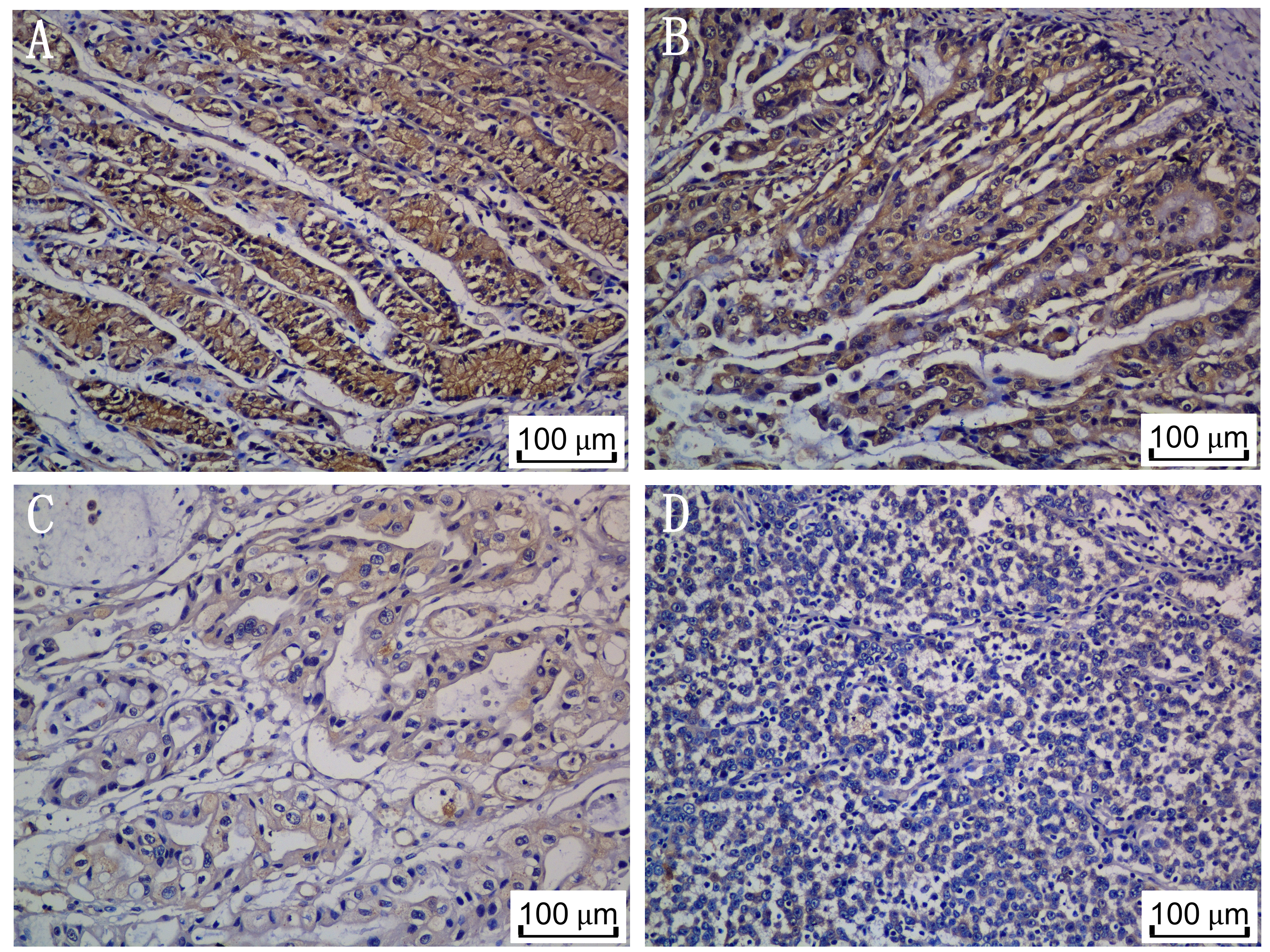

Supplement: Supplementary file 1 — Additional file 1. Additional table and figures. [file 40880_2019_347_MOESM1_ESM.doc]
